# The use of a biocompatible carbon fiber implant for cervical and lumbar spine fusion in the treatment of spondylodiscitis: a case report

**DOI:** 10.1093/jscr/rjag272

**Published:** 2026-04-17

**Authors:** Neha Aftab, Ricardo Alonso Catalan-Lopez, Arman Sourani, Christian Bowers, Marc D Moisi

**Affiliations:** Hurley Medical Center, Flint, MI, United States; Bowers Neurosurgery and Frailty Outcomes Data Lab, MI, United States; Michigan State University, Flint, MI, United States; Bowers Neurosurgery and Frailty Outcomes Data Lab, MI, United States; Bowers Neurosurgery and Frailty Outcomes Data Lab, MI, United States; Hurley Medical Center, Flint, MI, United States; Bowers Neurosurgery and Frailty Outcomes Data Lab, MI, United States; Michigan State University, Flint, MI, United States; Hurley Medical Center, Flint, MI, United States; Bowers Neurosurgery and Frailty Outcomes Data Lab, MI, United States; Michigan State University, Flint, MI, United States

**Keywords:** vertebral osteomyelitis, carbon fiber implant, spondylodiscitis, epidural abscess, epidural phlegmon

## Abstract

Osteomyelitis and discitis are rare conditions that predominantly affect immunocompromised patients. Surgical intervention is generally reserved for patients who do not respond to medical therapy or who present with spinal instability, extensive tissue destruction or abscess formation. Carbon fiber implants constitute a notable advancement in the management of these conditions, providing high biocompatibility, radiolucency that facilitates postoperative imaging, a favorable strength-to-weight ratio, enhanced bone healing, reduced complication rates, and a modulus of elasticity comparable to cortical bone. We report the case of a 62-year-old Caucasian male with insulin-dependent diabetes and neuropathy, diagnosed with discitis at C4–5, C5–6, L3–4, and L4-L5, as well as an anterior epidural phlegmon resulting in central canal narrowing. The patient underwent anterior cervical discectomy, spinal cord decompression, evacuation of the epidural abscess, placement of interbody carbon fiber cages at C4–5 and C5–6, anterior plate and screw fixation from C4 to C6, and subsequent surgery for L2-S1. This case report evaluates the application of biocompatible carbon fiber implants in the treatment of osteomyelitis and discitis, highlighting their potential advantages for clinical practice.

## Introduction

Vertebral osteomyelitis, defined as infection of the vertebral body, represents a serious condition that may result in significant neurological complications. This pathology frequently occurs in conjunction with discitis; when both the vertebral body and intervertebral disc are infected, the condition is referred to as spondylodiscitis. The diagnosis of spondylodiscitis requires involvement of two adjacent vertebrae and intervertebral discs [1]. Although spondylodiscitis can affect individuals of all ages, those who are immunocompromised or have chronic conditions such as renal failure, diabetes mellitus, cancer, or a history of organ transplantation or intravenous drug use are at increased risk [2]. The standard treatment for spondylodiscitis is conservative management with antibiotics. Surgical intervention is reserved for cases involving mechanical instability, epidural abscess, spinal cord compression, neural irritation, neurological deficits, failure of conservative therapy, or septic complications [3].

Disease severity and pre-existing spinal pathology are critical factors in determining the necessity for neurosurgical intervention. This report describes a 62-year-old male with osteomyelitis discitis who underwent surgical management. The patient received a C4–C6 anterior cervical plate with carbon fiber screws. Additional procedures included lumbar lateral interbody fusion at L3–4 and L4–5, L4-S1 laminectomy for abscess drainage, and L2-S1 fusion. Carbon fiber pedicle screws (CFPS) have recently emerged as an alternative to titanium for the treatment of fractures and primary or metastatic spinal tumors. Recent studies have begun to document their application in spondylodiscitis.

## Case presentation

A 62-year-old Caucasian male with uncontrolled insulin-dependent diabetes, neuropathy, and chronic alcohol use presented with generalized weakness and a history of diabetic foot wound, multiple falls, raising suspicion for necrotizing infection. He reported neck pain radiating to both upper extremities with associated paresthesias, as well as chronic lower back pain radiating to the lower extremities.

**Figure 1:**
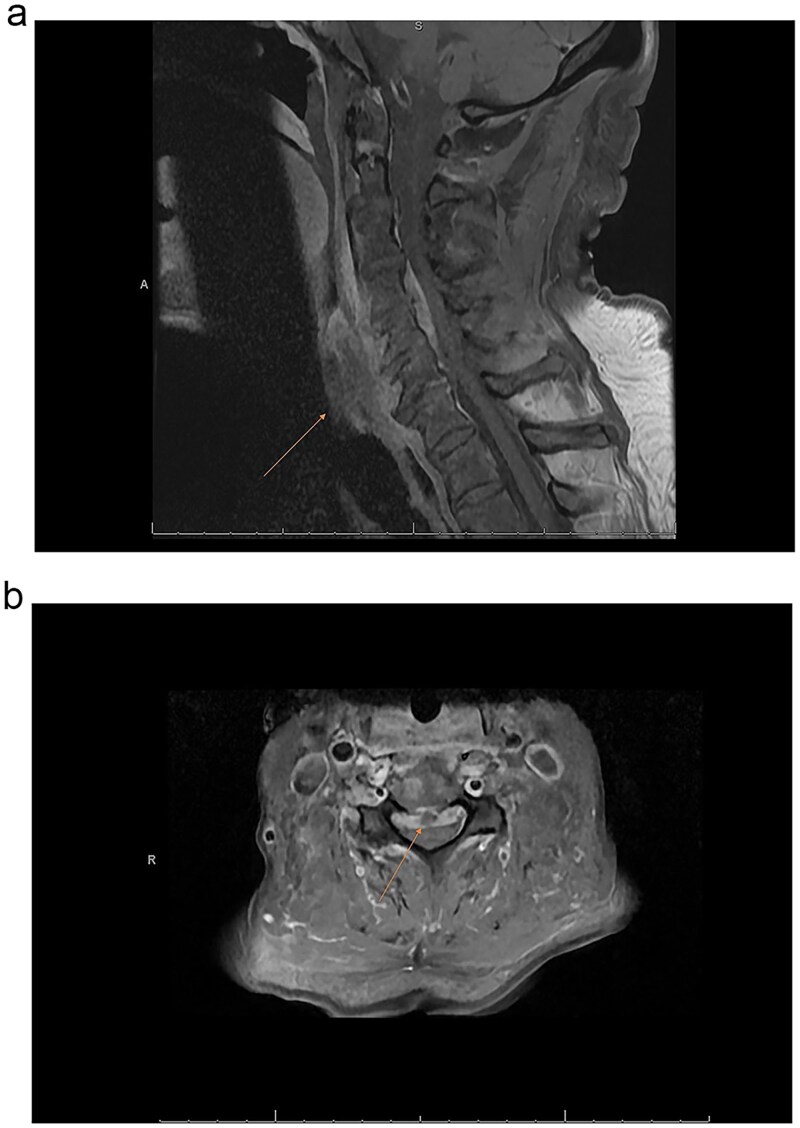
(a) MRI cervical sagittal of T1 vertebrae with gadolinium contrast depicting osteomyelitis/discitis with epidural abscess. (b) MRI cervical axial T1 image with gadolinium contrast C4–5 demonstrating epidural abscess and cord compression.

**Figure 2:**
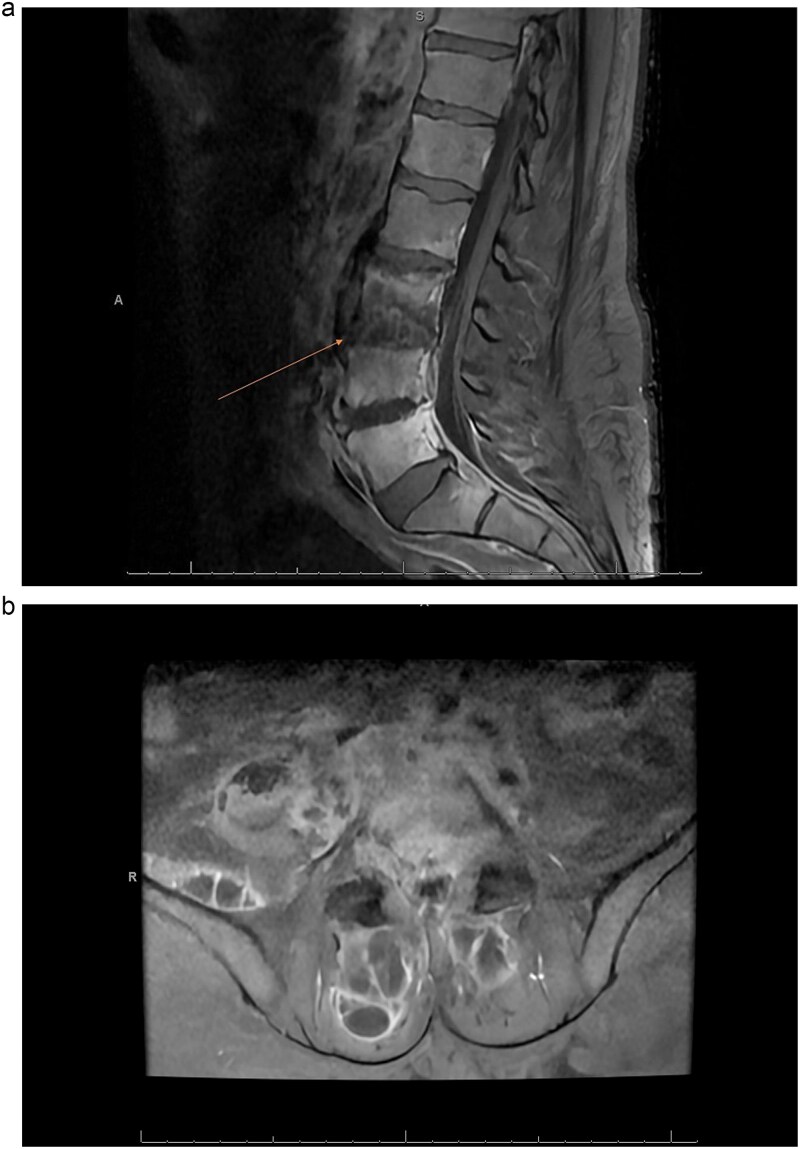
(a) MRI sagittal lumbar T1 with gadolinium contrast showing osteodiscitis at L3–4 and L4–5 vertebrae with epidural abscess. (b) Axial lumbar MRI T1 with gadolinium contrast showing epidural abscess with iliopsoas and paraspinal abscesses.

Magnetic resonance imaging (MRI) of the spine demonstrated discitis at C4–5 and C5–6. Imaging further revealed an enhancing anterior epidural phlegmon extending from C3–4 to C5–6, with central canal narrowing at C4–5 (Fig. 1a and b). Additional findings included osteomyelitis of the L3–4 and L4–5 vertebral bodies, bilateral iliopsoas muscle abscesses, and abscesses posterior to the L5 and S3 vertebral bodies (Fig. 2a and b). The surgical plan consisted of anterior cervical discectomy and fusion. The procedures performed included C4–5 and C5–6 anterior cervical discectomy with spinal cord decompression and evacuation of epidural abscess, C4–5 and C5–6 anterior interbody arthrodesis, and insertion of polyetheretherketone (PEEK) interbody biomechanical cages. An anterior cervical carbon fiber plate and screws (Icotec) were placed from C4 to C6.

**Figure 3:**
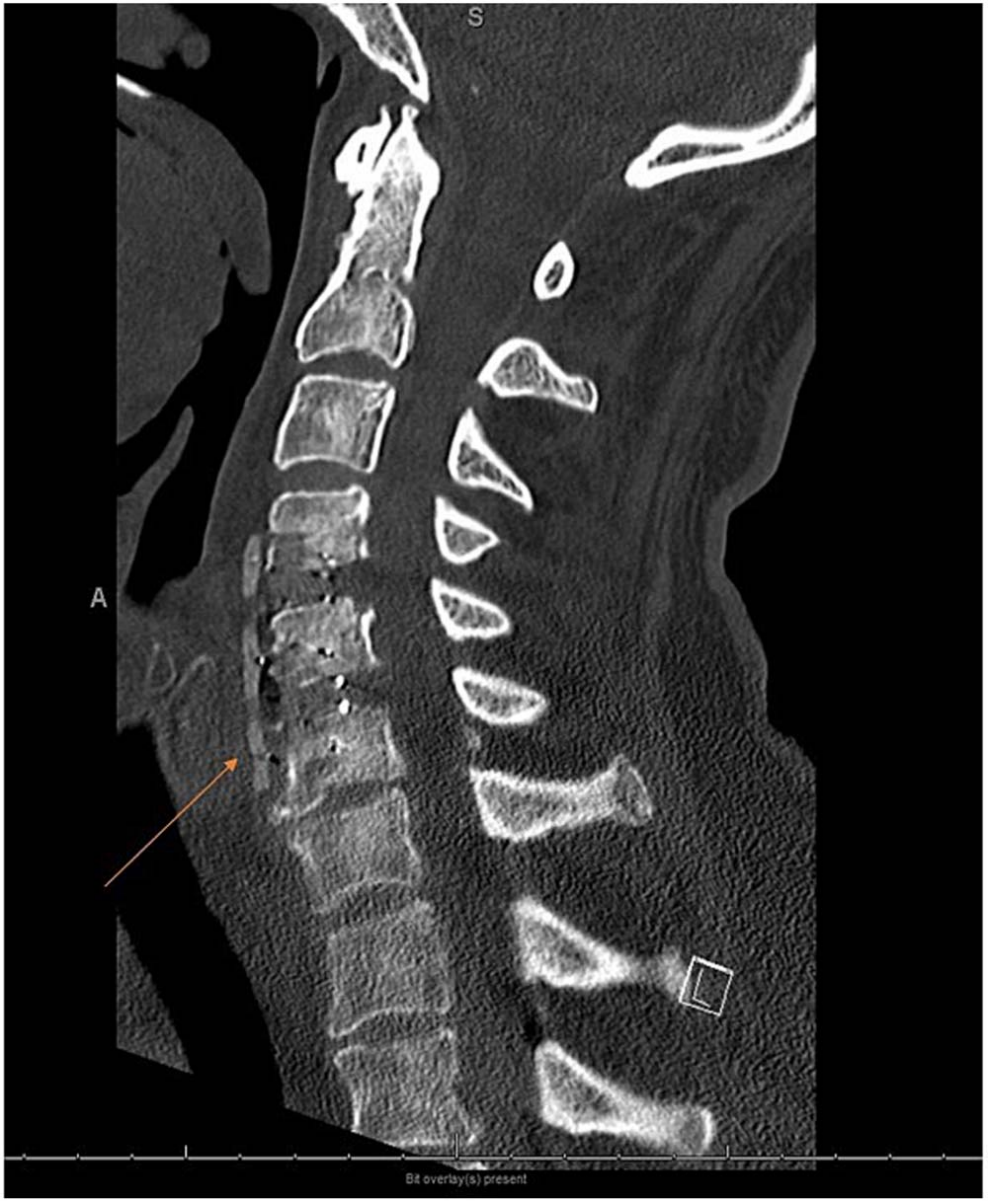
Postoperative sagittal cervical CT scan image sagittal view showing a carbon fiber plate with screws.

**Figure 4:**
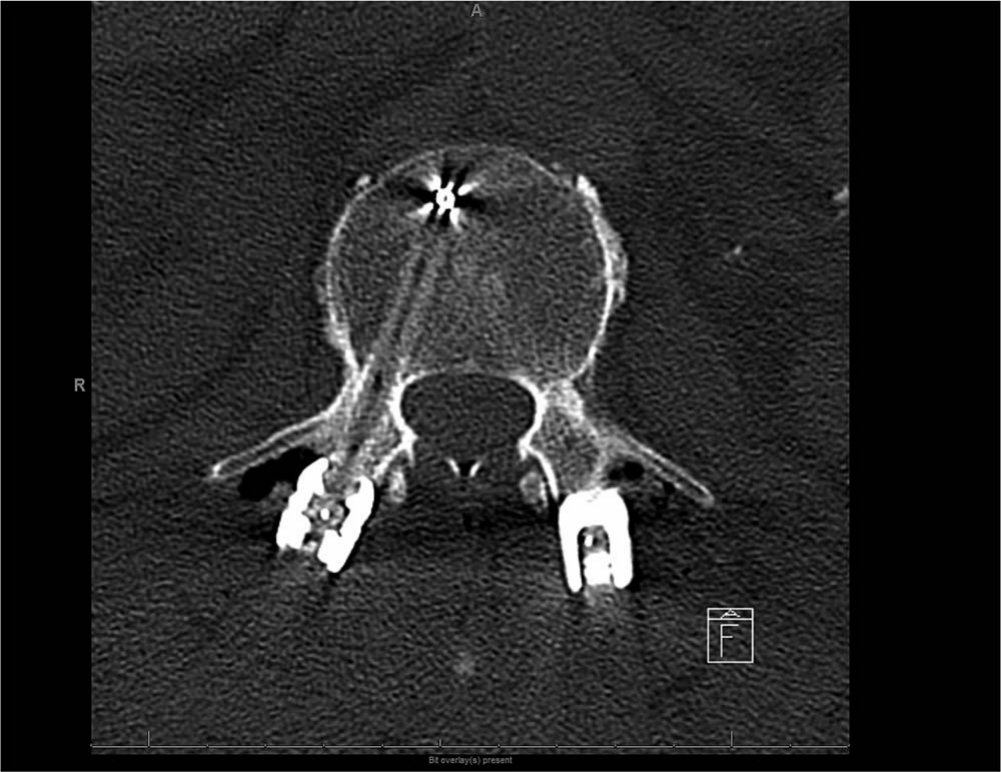
Postoperative CT scan lumbar axial view showing a carbon fiber screw with no visible scatter.

**Figure 5:**
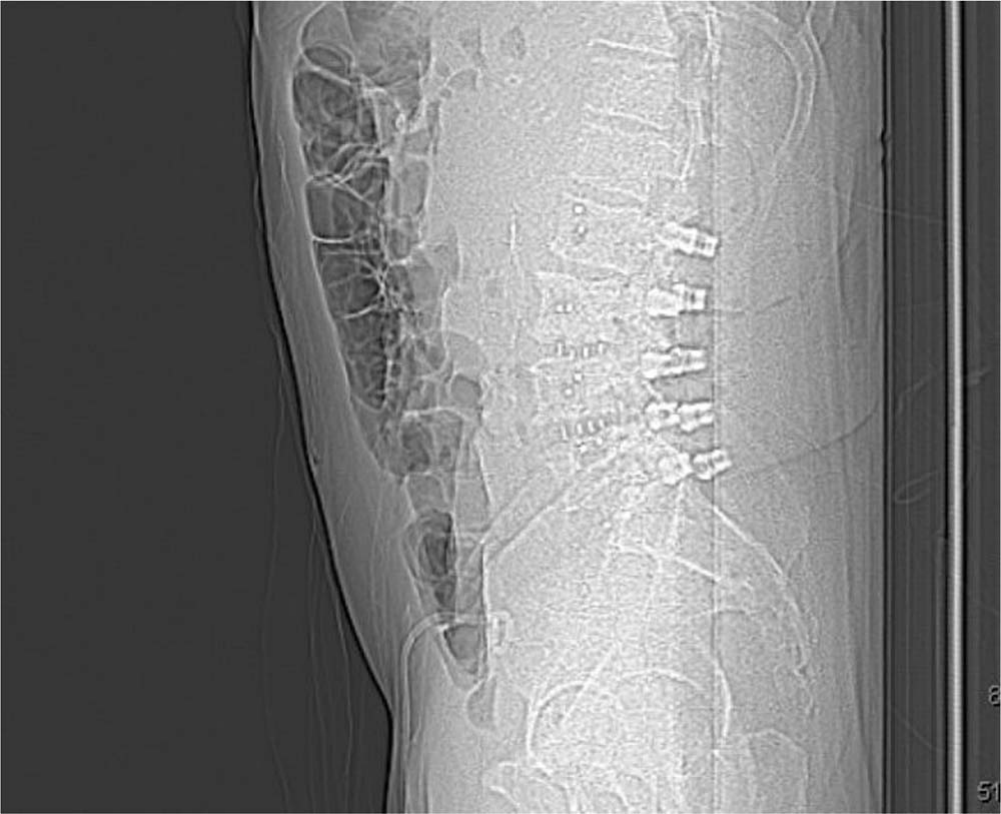
Postoperative sagittal X-ray demonstrating a carbon fiber plate and screws (lateral view).

**Figure 6:**
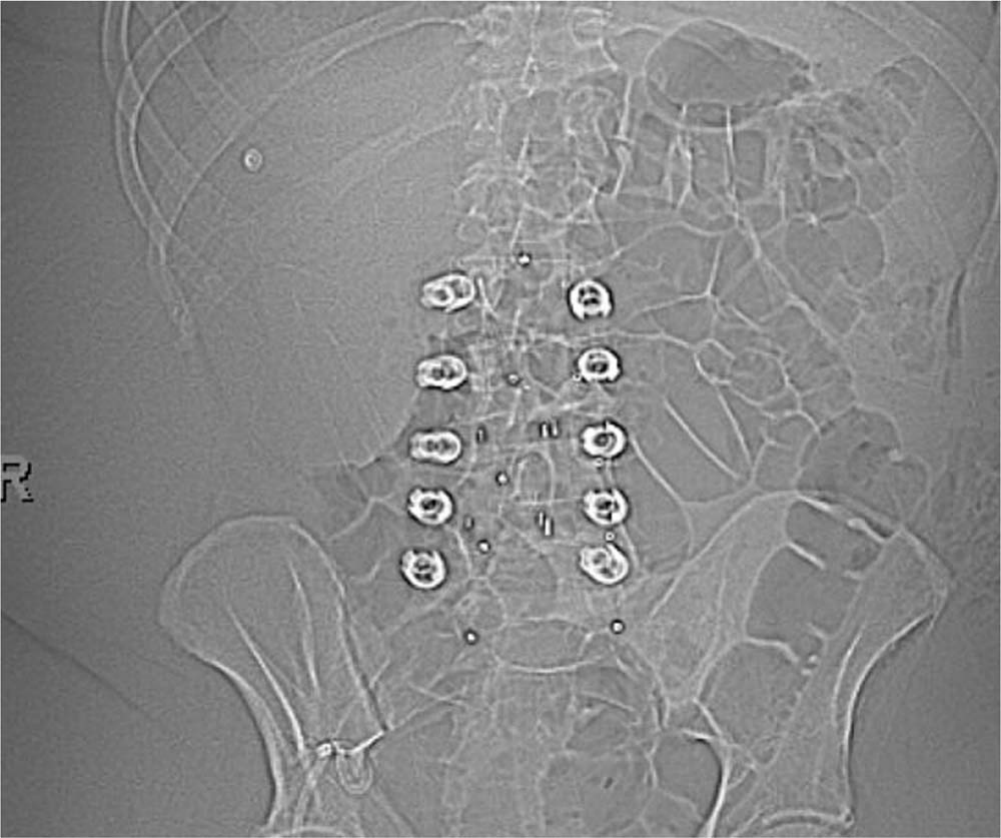
Postoperative A-P X-ray showing carbon fiber plate and screws. (AP view).

The patient was transferred to the intensive care unit (ICU) with a Miami-J collar in place. Intraoperative cultures were positive for Staphylococcus, prompting initiation of antibiotic therapy. Post-operative computed tomography (CT) imaging demonstrated preserved alignment at C4-C5 and C5-C6. The patient exhibited significant improvement in knee flexion, knee extension, and ankle flexion following surgery. He subsequently underwent lumbar lateral interbody fusion at L3–4 and L4–5, followed by lumbar L4–5 hemilaminectomy, L5-S1 laminectomy, L2-S1 posterior instrumentation with pedicle screws and rods, and arthrodeses at L2–L3, L3-L4, L4-L5, and L5-S1 (Figs 3–6). Given the history of multiple spinal osteomyelitis and epidural abscesses, a fluid collection developed from L1-L2 through S1, raising concern for abscess formation. Incision and debridement of the lumbar wound were performed, and a superficial wound vacuum-assisted closure (VAC) was applied. Wound cultures grew *Serratia marcescens*, *Proteus vulgaris*, and Gram-negative rods. The patient was initially started on metronidazole and cefepime empirically; following culture results, treatment was changed to daptomycin and ertapenem for a total of 6 weeks.

## Discussion

Early surgical intervention outperforms conservative management in mortality rates and length of stay in patients with spondylodiscitis [3]. Spinal fusion is widely regarded as a cornerstone of surgical management for spondylodiscitis. Several studies have demonstrated that spinal fusion provides sustainable clinical outcomes with numerous benefits [4]. Several factors must be considered when selecting an instrument. Metallic screws have been broadly used in spinal surgeries, including those for discitis. Titanium screws, in particular, are well-established, with osseointegration rates exceeding 80% and favorable biomechanical properties. However, metallic implants can interfere with postoperative CT and MRI imaging, resulting in significant artifacts, such as beam-hardening effects resulting in poor postoperative visualization when most critical for decision making [4, 5].

PEEK implants have become a standard interbody cage material in spine surgery due to their minimal artifact production, favorable biocompatibility, radiolucency, and cost-effectiveness. CFPS, specifically carbon fiber-reinforced PEEK (CFR-PEEK) screws, offer greater mechanical strength than PEEK implants and have recently emerged as alternatives to metallic implants in spine oncology and in spondylodiscitis. These screws not only combine the advantageous properties of PEEK, but also have an elastic modulus similar to human cortical bone [6, 7]. CFPS are biocompatible, chemically resistant, non-ferromagnetic, and highly radiolucent, which minimizes interference with CT and MRI imaging. Biomechanical studies indicate that CFPS provide strength, pullout resistance, bending force, and stiffness comparable to titanium screws. Furthermore, their mechanical stress distribution closely resembles that of cortical bone, which may reduce the risk of hardware failure [8].

The use of CFPS has been indicated in several spinal pathologies, including primary and spinal metastatic diseases. However, their use in spondylodiscitis remains limited, and only a few studies evaluate the efficacy, applicability, safety, and outcomes of CFPS. We report a case of cervical and lumbar spondylodiscitis successfully treated using a combined anterior and posterior approach with carbon-fiber-reinforced instrumentation, resulting in excellent clinical outcomes.

Advancements in implant materials have created a more biocompatible environment for patients. Carbon fiber implants offer several key advantages, primarily due to their radiolucency. This characteristic facilitates easy visualization, minimizing imaging artifacts compared to metallic implants [7, 9]. This is particularly beneficial for postoperative monitoring, tumor surveillance, and precise radiation planning [9]. This can be translated to infection of the spine due to the inherent need for good margins to follow the disease process.

Furthermore, carbon fiber’s elastic modulus (~3.5 GPa), while differing significantly from cortical bone (12–20 GPa), is closer to that of stainless steel (230 GPa) or titanium (106–155 GPa) [9]. This similarity reduces stress concentrations at the implant-bone interface [9]. Despite this difference, carbon fiber implants effectively withstand high-strain loads [7]. Compared with titanium implants, carbon fiber implants significantly reduce postoperative imaging artifacts, beam perturbation, and dose degradation during radiation therapy [7]. These combined properties enhance bone healing, minimize the risk of infection, and reduce the risk of implant failure. In a retrospective study of 81 consecutive patients who underwent CFR PEEK implants for spondylodiscitis, Burkhardt *et al.* reported ideal MRI reportability of soft tissue structures, improved mobility, and reduced pain [10]. As this case report focuses on a single patient, the generalizability of the findings is limited and the findings are subject to selection bias. While postoperative improvement was observed on imaging, long-term follow-up data is being collected to evaluate potential complications. Nevertheless, the report offers valuable insights into the application of carbon fiber implants in the treatment of spondylodiscitis.

## Conclusion

This report describes a case of spondylodiscitis managed with CF-PEEK implants as an alternative to titanium. Previous studies have demonstrated that CF-PEEK implants are equivalent to, and potentially superior to, titanium implants for spinal applications. This case demonstrates improved post-operative imaging visibility, which facilitates enhanced assessment of the disease process during healing. Enhanced visualization allows for clearer differentiation between residual or recurrent disease and completion of the treatment process. Additionally, this case underscores the need for further comparative prospective studies in patients with spondylodiscitis.
